# Aging-dependent decrease in the numbers of enteric neurons, interstitial cells of Cajal and expression of connexin43 in various regions of gastrointestinal tract

**DOI:** 10.18632/aging.101677

**Published:** 2018-12-11

**Authors:** Tingyi Sun, Dandan Li, Shilong Hu, Li Huang, Haimei Sun, Shu Yang, Bo Wu, Fengqing Ji, Deshan Zhou

**Affiliations:** 1 Department of Histology and Embryology, School of Basic Medical Sciences, Capital Medical University, Beijing 100069, China; 2 Beijing Key Laboratory of Cancer Invasion and Metastasis Research, Beijing 100069, China; 3 Cancer Institute of Capital Medical University, Beijing100069, China

**Keywords:** aging, gastrointestinal motility, interstitial cells of Cajal, enteric nervous system, connexin43

## Abstract

Aging is a significant risk factor for gastrointestinal dysmotility, but aging-associated differences between different organs and the exact time to start degenerating have remained obscure. Here we evaluated alterations of interstitial cells of Cajal, enteric neurons and connexin43 expression in the stomach, jejunum and colon in 2-, 12-, 16-, 20- and 24-month-old mice, as well as in aged human colon. Interstitial cells of Cajal, cholinergic and nitrergic neurons within the whole digestive tract were reduced over time, but their loss first appeared in stomach, then in intestine, helping to understand that gastric function was first impaired during aging. The decrease of connexin43 expression occurred before interstitial cells of Cajal and neurons loss, suggesting that connexin43 might be the major target influenced during senescence. Furthermore, changes in expressions of pro-inflammatory cytokines (tumour necrosis factor-α, interleukin-1β, interleukin-6) and apoptosis-related proteins (B-cell lymphoma-2, caspase-3) which indicated “inflammaging”, might contribute to the loss of enteric neurons and interstitial cells of Cajal in aged gastrointestinal tract. Our results provide possible therapeutic time window for beneficial intervention for geriatric patients with gastrointestinal motility disorders.

## Introduction

As the aging population increases worldwide, gastrointestinal (GI) dysfunction is becoming a notable social problem. GI motility disorders, in particular, are prevalent amongst the elderly, and cause significant physical, emotional, and financial burdens due to chronic constipation, fecal incontinence, even GI tumors. Among them, the incidence of constipation is striking, about 20% of old people experience chronic constipation, which results in failure to absorb nutrients and eliminate harmful metabolites, as well as increasing incidence of infection, and so being involved in the etiology of GI diseases [1,2]. It is believed that the deeper understanding on the pathological proceeding of GI motility disorders will provide a special “window of opportunity” for the early intervention and delay disease progression in the elderly.

It is well known that rhythmic peristalsis is essential for a range of vital functions, such as digestion, absorption, secretion and excretion. This motility has been convincingly shown to require the normal distribution and function of interstitial cells of Cajal (ICCs), the pacemaker cells in the GI tract, which generate spontaneous slow electrical waves and ultimately lead to concentric smooth muscle contraction allowing propulsion of intestinal contents [3,4]. Loss of ICCs is frequently associated with several human GI motility disorders like diabetic gastroparesis [5], Hirschsprung’s disease [6], slow transit constipation [7], and achalasia [8]; and data from Gomez-Pinilla’s study demonstrated that age-associated decline in ICCs number and network do occur in the normal human stomach and colon [9], exploring the potential role of ICCs in the onset and development of GI disorders during senescence. Even so, little has been thoroughly documented whether the decrease of ICCs performs synchronously at different segments of alimentary canal or the accurate age at which number of ICCs starts to reduce.

A number of studies, based upon the structural and functional relationship between ICCs and enteric nerve fibers, have strongly suggested the role of ICCs in neurotransmission of signals from enteric neurons to smooth muscle cells (SMCs), i.e., the afferent neural transduction through ICCs [4,10]. Moreover, neurons can also directly innervate GI SMCs [10]. The GI tract contains the largest number of neurons constituting enteric nervous system (ENS) outside the central nervous system to regulate gut normal physiologic functions [11]. The ENS is mainly composed of the submucosal plexus and myenteric plexus (MP), the latter which locates between longitudinal and circular smooth muscle layers, is involved with initiation and control of smooth muscle contraction and plays a crucial role in the regulation of peristalsis [11]. Enteric neurodegeneration is regarded as a pathophysiologic foundation of digestive manifestations in certain disease states, and GI dysfunction in the neurodegenerative disorders is related, at least in part, to abnormalities of excitatory cholinergic neurons and inhibitory nitrergic neurons within ENS [12,13]. Much of the research has proved that the neuronal loss contributes to age-associated GI dysfunction [14,15]. According to the complex network of ENS, the illustration of the initial time point of neuronal degeneration in the GI tract could therefore be helpful to the gerontology research. For instance, older people have greater distension of gastric antrum and slower gastric emptying than those of young adults [16,17], but the underlying mechanism has remained obscure.

Apart from ICCs and ENS, normal GI motility function is also coordinated by the intercellular signal communication, which is typically governed by gap junctions [18]. The connexin43 (Cx43), which widely exists between ICCs or ICCs and SMCs, is the most important protein of gap junctions in mediating synchronized contraction of SMCs and ICCs function [18,19]. Studies of Hirschsprung’s disease over the past decades have exhibited that lack of Cx43-based communication between ICCs or/and SMCs may be partly responsible for the GI motility disorders [20,21]. McClain et al. [22] showed that Ca^2+^ responses in enteric glia, mediated by Cx43 hemichannels, influenced gut motility and intestinal transit in mice. Besides, Cx43 also has close relations with spontaneous beating of cardiomyocytes in cardiac conduction system [23]. Given the above, we can reasonably surmise that Cx43 might take part in maintaining the GI function, but alteration of Cx43 involved in age-related dysmotility is far from certain.

Therefore, the present study aims to elucidate the morphological alterations and quantitative assessment of enteric neurons including cholinergic and nitrergic neurons as well as its surrounding ICCs in the MP of mice aged 2, 12, 16, 20 and 24 months (mo). We also observed Cx43 protein expression in longitudinal muscle layer with the adherent MP and ICCs of young and old mice. All results were further validated in aged human colon. Our study demonstrated that the sparseness or absence of ICCs and enteric neurons, which first appeared in stomach and partially caused by inflammation, could contribute to the age-associated GI motility dysfunction. And the decrease of Cx43 expression occurred before ICCs and neurons loss in the GI tract.

## RESULTS

### Intestinal transit in mice is significantly delayed with time

**Figure 1:**
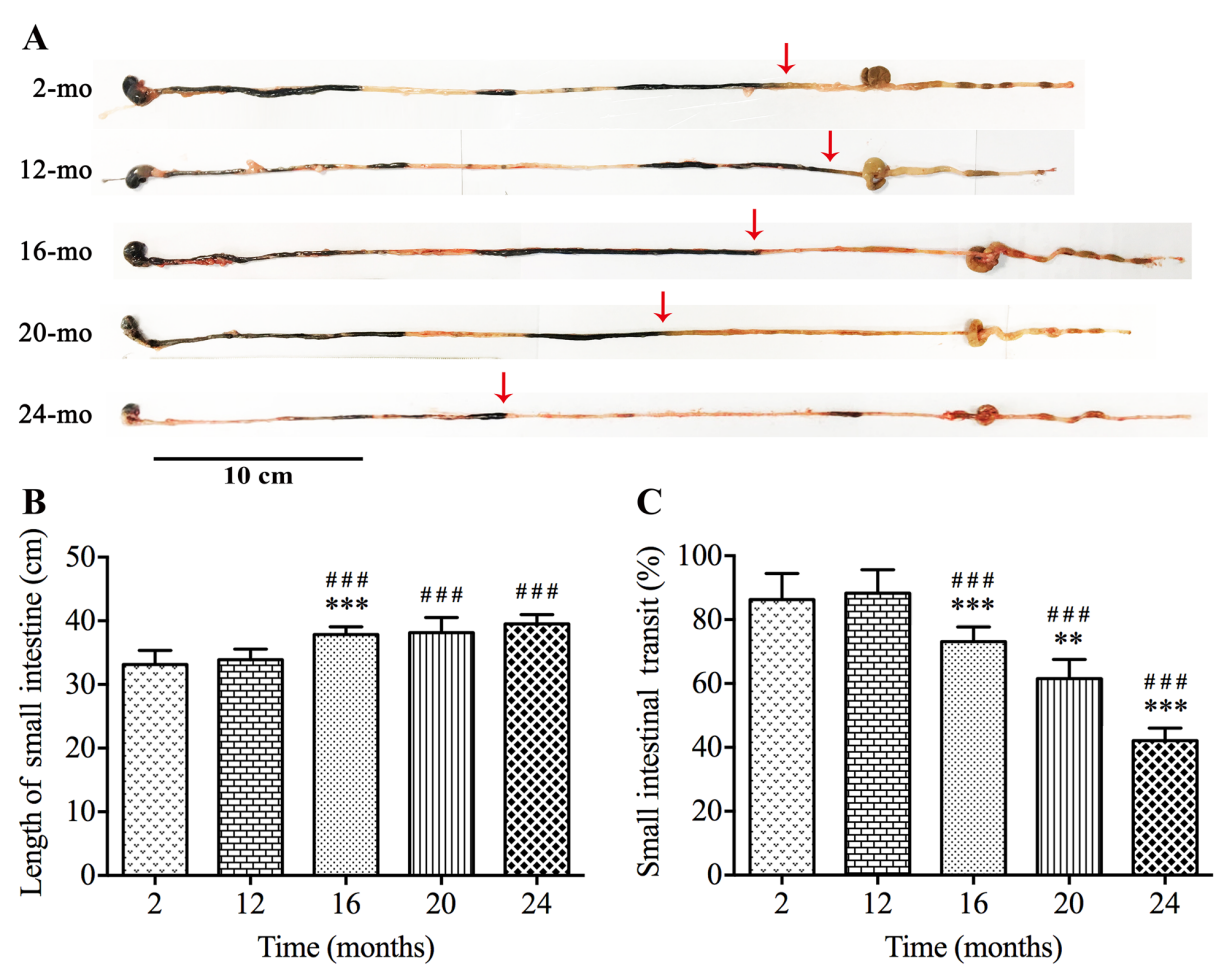
**Slower intestinal transit during aging in mice.** The ink propulsion distance was illustrated in different age groups (**A**). The length of small intestine was extended (**B**) while *in vivo* intestinal propulsion rate was reduced (**C**) during aging. Statistical analysis was performed using one-way analysis of variance and data were represented as mean ± SD, statistical significance is: ### *P* < 0.001 compared with 2-mo-old group; ** *P* < 0.01, *** *P* < 0.001 compared with previous group; n=8 mice per group.

To evaluate the GI motility function during aging *in vivo*, we used intestinal transit rate as a concernful parameter. The transit of the carbon marker 30 minutes after gavage was restricted to the small intestine in different groups (Fig. 1A). The ink propulsion distance of 20- and 24-mo-old mice was markedly shorter than that of younger groups, but no remarkable difference was observed between mice from 2 mo to 16 mo. We also found that the length of the entire small intestine was added from 16 mo (Fig. 1B). Hence the transit rate was reduced from 88.25% in 12-mo-old mice to 42.13% in 24-mo-old mice (Fig. 1C).

### ICCs in the mouse GI tract decrease with increasing age

**Figure 2:**
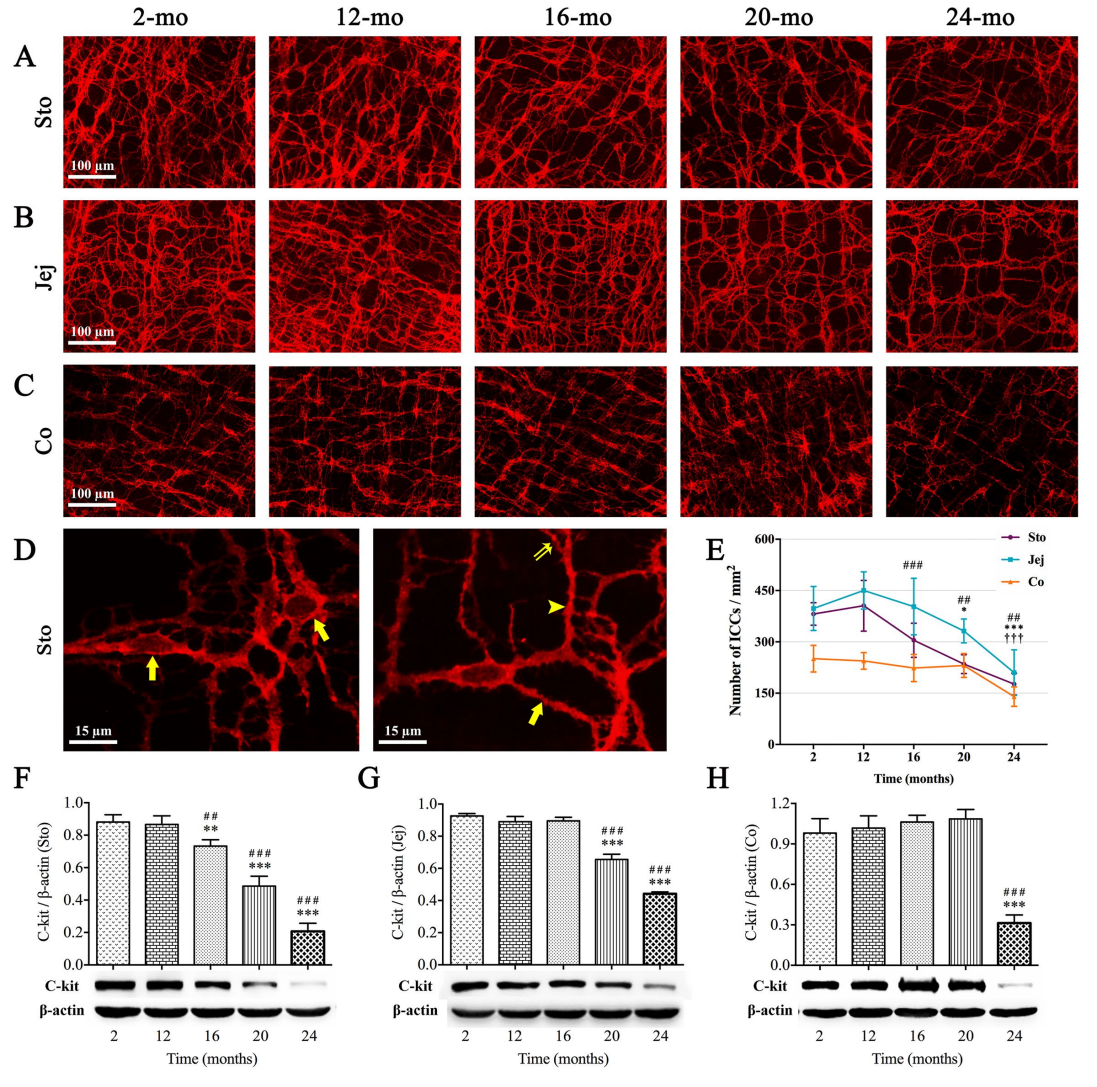
**Influence of aging on ICCs in the mouse GI tract.** The c-kit immunoreactivity (red) showed ICCs network in the whole-mount preparation. The sparseness of cellular network in stomach (**A**), jejunum (**B**) and colon (**C**) appeared at 16, 20 and 24 mo, respectively. In high magnification of 2-mo-old stomach (**D**), c-kit(+) cells with round or oval cell bodies (left figure, arrows) and cell processes (right figure) including primary (arrow), secondary (arrowhead) and tertiary processes (double arrow) were clearly seen by c-kit immunofluorescence staining. Statistical analysis showed that ICCs density decreased over time from 16 mo in stomach, 20 mo in jejunum and 24 mo in colon (**E**). Expressions of c-kit protein in 2-, 12-, 16-, 20- and 24-mo-old mice in different organs of GI tract were examined by western blotting (**F-H**). Densitometric analysis of protein expressions normalized to β-actin and the downtrend of c-kit expression was coincident with ICC-density in all three organs. Statistical analysis was performed using one-way analysis of variance and data were represented as mean ± SD, statistical significance is: (**E**) ## *P* < 0.01, ### *P* < 0.001 compared with previous stomach group; * *P* < 0.05, *** *P* < 0.001 compared with previous jejunum group; ††† *P* < 0.001 compared with previous colon group; (**F-H**) ## *P* < 0.01, ### *P* < 0.001 compared with 2-mo-old group; ** *P* < 0.01, *** *P* < 0.001 compared with previous group; n=5 mice per group. Abbreviation: Sto, stomach; Jej, jejunum; Co, colon.

ICCs express the proto-oncogene c-kit related to the development and maintenance of ICCs [24]. The immunofluorescence staining (Fig. 2A-D) and western blot analysis (Fig. 2F-H) of c-kit showed that the proportions of ICCs in the MP region of the mouse GI tract including stomach, jejunum and colon all tended to decrease during aging. In the case of stomach, in 2-mo-old mice, the cell body of ICCs was big, their processes were thick and there were many secondary and tertiary branches, which interlaced and formed a complete network (Fig. 2D). As age increased from 16 mo, not only the number of ICCs in stomach was decreased, but also the processes presented fewer branches and cellular network was sparse. Like those in stomach, the similar tendency was also observed in either jejunum or colon, however, the alterations in ICCs appeared later considerably: it was at 20 mo in jejunum, and at 24 mo in colon (Fig. 2E). And age-related damage to cellular network was clearly observed in the colon of the oldest individuals (24-mo-old).

### The number of ENS neurons in the mouse GI tract decreases with aging

Immunofluorescence staining and NADPH-diaphorase (NADPH-d) histochemistry were used to label corresponding ENS neurons, respectively, in whole-mount preparations according to specific biomarkers as follows: (1) choline acetyltransferase (ChAT) for excitatory cholinergic neurons; and (2) nitric oxide synthase (NOS) for inhibitory nitrergic neurons.

**Figure 3:**
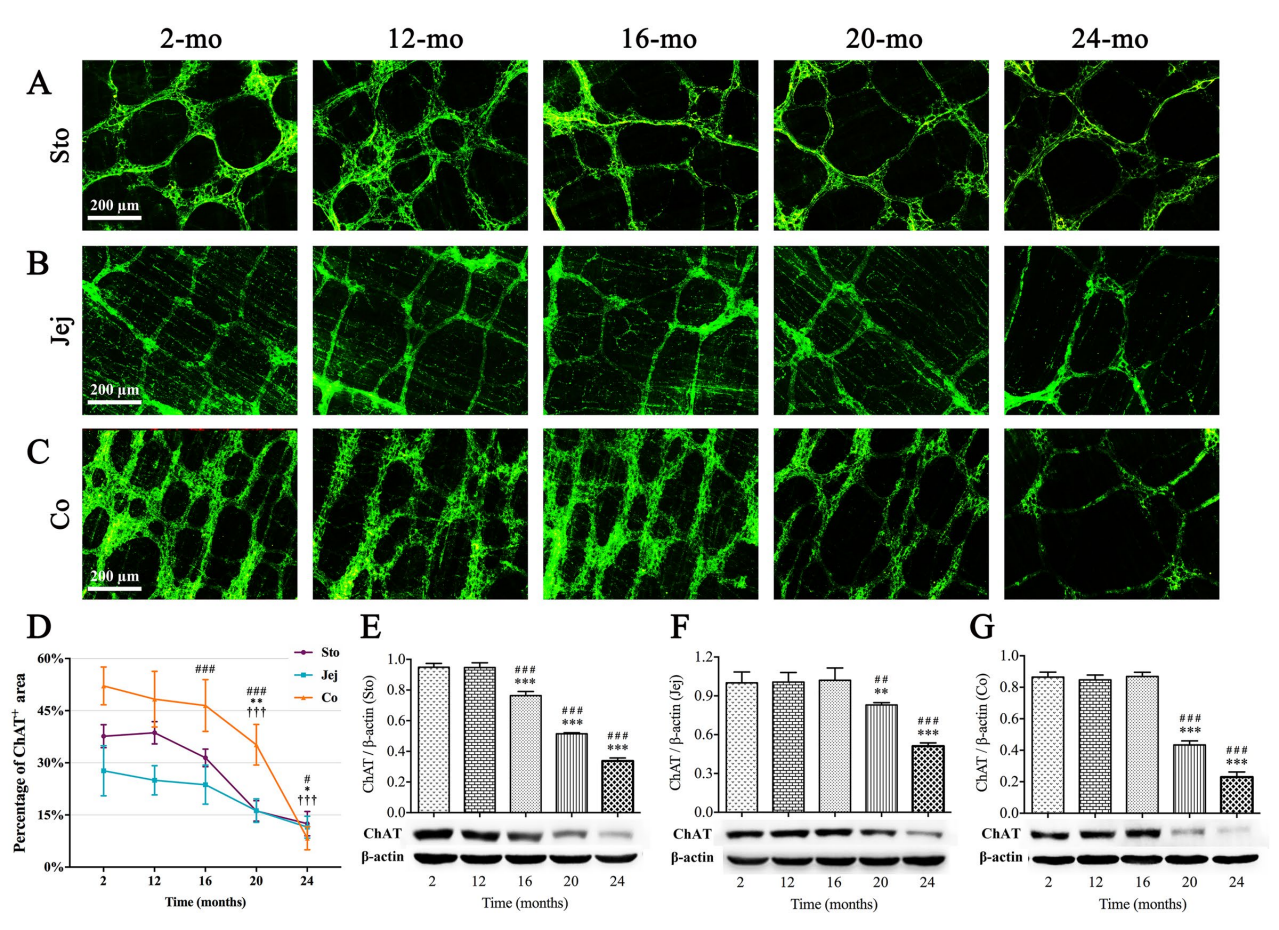
**Decrease in ChAT(+) neurons in the MP of mouse GI tract with aging.** ChAT immunoreactivity (green) was shown in ganglia and nerve ﬁbers. ChAT-positive area per field gradually decreased from 16 mo in stomach (**A**), 20 mo in jejunum (**B**) as well as 20 mo in colon (**C**), respectively. Diminished immunoreactive area density (**D**) and the decline in expression of ChAT protein (**E-G**) were observed in aging mice consistent with morphological results. Densitometric analysis of protein expressions normalized to β-actin. Statistical analysis was performed using one-way analysis of variance and data were represented as mean ± SD, statistical significance is: (D) # *P* < 0.05, ### *P* < 0.001 compared with previous stomach group; * *P* < 0.05, ** *P* < 0.01 compared with previous jejunum group; ††† *P* < 0.001 compared with previous colon group; (E-G) ## *P* < 0.01, ### *P* < 0.001 compared with 2-mo-old group; ** *P* < 0.01, *** *P* < 0.001 compared with previous group; n=5 mice per group. Abbreviation: Sto, stomach; Jej, jejunum; Co, colon.

ChAT is the rate-limiting enzyme that is required for the acetylcholine synthesis. Strong immunoreactivity for ChAT (green) was obviously seen in the MP of GI tract, including neurons with their primary strands, secondary bundles and ﬁne tertiary ﬁbers. In young mice, several distinct round or ovoid ChAT-positive neurons were located within each ganglion, and the granular positive reactant was clearly scattered around the cell body and also within interganglionic nerve bundles. The neural networks became sparse in old mice indicating the senescence phenomenon also appeared in the ENS of the mouse GI tract (Fig. 3A-C). According to statistical analysis, compared with young (2-mo-old) and middle-aged (12-mo-old) mice, ChAT-positive area of ganglia and nerve ﬁbers per field was gradually decreased in the stomach from 16 mo; however, reduction of ChAT(+) area was started at 20 mo of age in the intestine (Fig. 3D). The similar results were obtained by western blotting (Fig. 3E-G).

**Figure 4:**
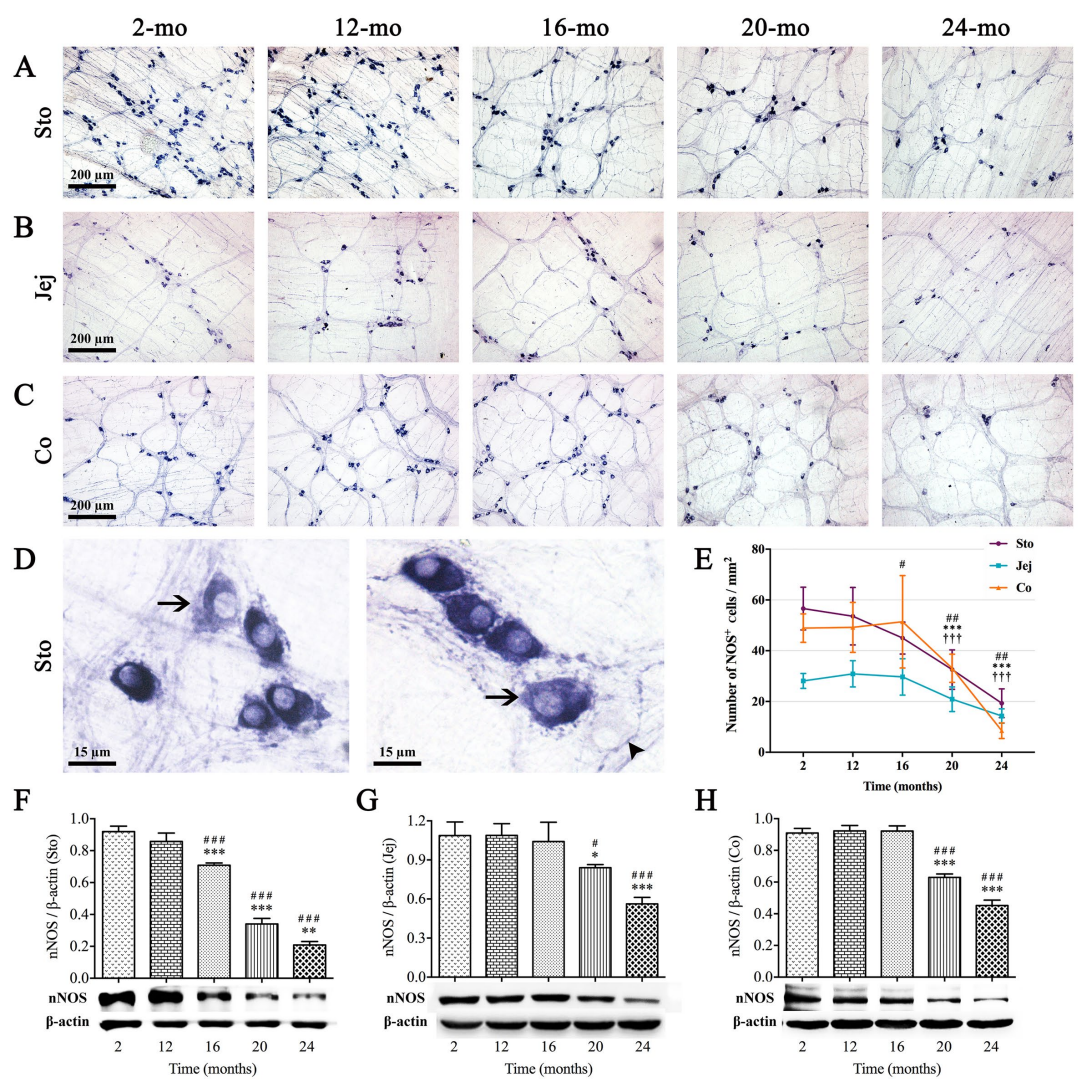
**Age-related reduction in nitrergic neurons in the MP of mouse GI tract.** Whole-mount preparations stained with NADPH-d histochemistry showed the variations in number of nitrergic neurons in stomach (**A**), jejunum (**B**) and colon (**C**) which appeared at 16, 20 and 20 mo, respectively. In higher magnification of 2-mo-old stomach (**D**), more typical NOS(+) neurons were oval in shape and intensely stained, with unstained nuclei. Some lighter cell bodies of NOS(+) neurons (left and right figures, arrows) were also seen which suggested that the activity of NOS were lower. Few cells with vaguely remained cellular outlines (right figure, arrowhead) shown in ganglia were confirmed to be other types of neurons. Statistical analysis showed that the decrease of NOS(+) neuronal numbers was started from 16 mo in stomach, 20 mo in jejunum and colon (**E**). Western blotting indicated that the trend of nNOS expression was coincident with the number of NOS(+) neurons in all three organs (**F-H**), and densitometric analysis of protein expressions normalized to β-actin. Statistical analysis was performed using one-way analysis of variance and data were represented as mean ± SD, statistical significance is: (E) # *P* < 0.05, ## *P* < 0.01 compared with previous stomach group; *** *P* < 0.001 compared with previous jejunum group; ††† *P* < 0.001 compared with previous colon group; (F-H) # *P* < 0.05, ### *P* < 0.001 compared with 2-mo-old group; * *P* < 0.05, ** *P* < 0.01, *** *P* < 0.001 compared with previous group; n=5 mice per group. Abbreviation: Sto, stomach; Jej, jejunum; Co, colon.

NADPH-d histochemistry is believed to be a good method for quantifying numbers of nitrergic neurons, which represented one of subpopulation of enteric neurons. Intriguingly, the changes of NOS(+) neurons in different organs of GI tract were shown in coincidence with ChAT-positive neurons. NOS(+) neurons were small and very intensely stained, with clearly visible unstained nuclei; meanwhile, there were a few labelled cell bodies scattered within nerve fibers (Fig. 4A-D). Alterations of total numbers of NOS(+) neurons per unit area (mm^2^) with age were shown in Fig. 4A-C and Fig. 4E, respectively. A signiﬁcant reduction in NOS(+) neuron number per mm^2^ was shown in stomach between 12- and 16-mo-old mice, but no reduction in jejunum and colon until 20 mo. Furthermore, age-related expression of neuronal nitric oxide synthase (nNOS) protein, measured by western blotting, was markedly decreased in aging mice, which was consistent with the alteration of NOS(+) neurons (Fig. 4F-H).

**Figure 5:**
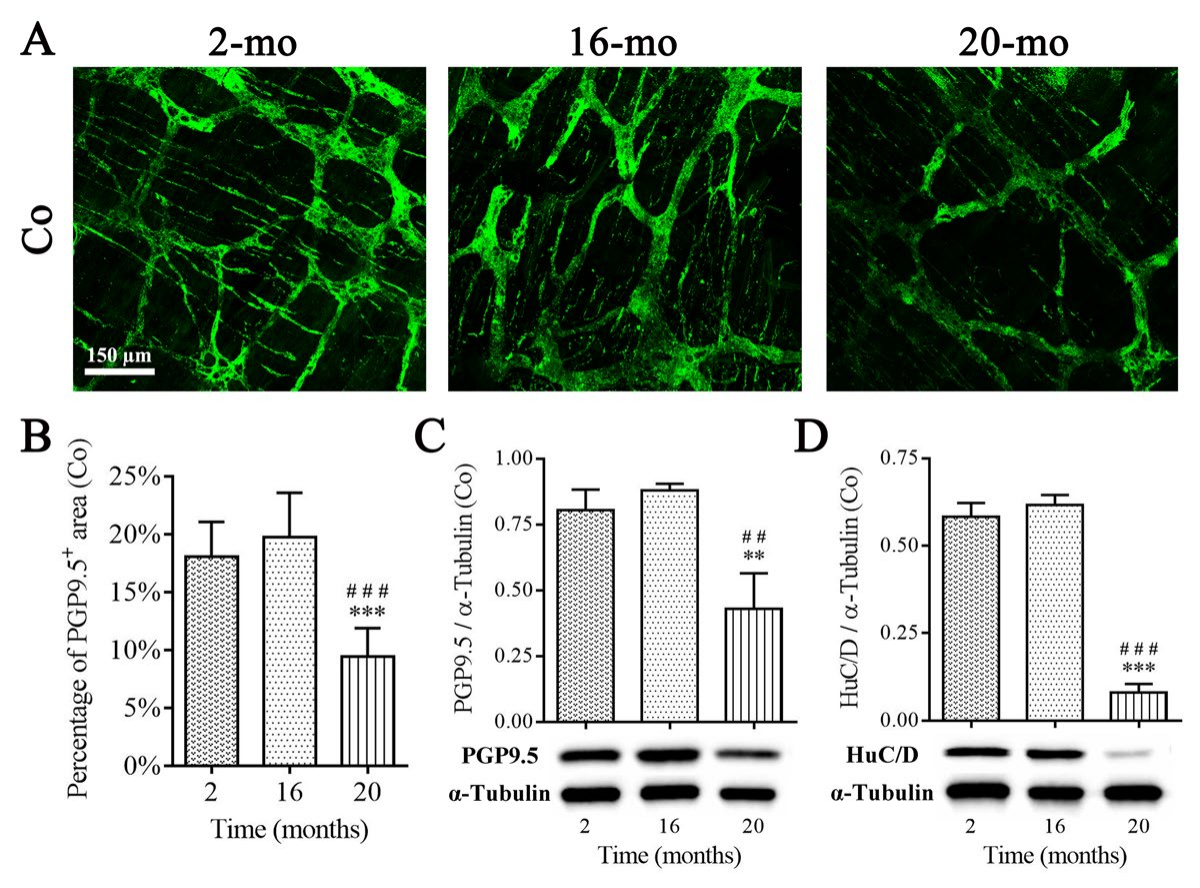
**Diminished enteric neurons in the colon of old mice.** PGP9.5 recognized perikarya and nerve fibers within MP, and PGP9.5-positive area per field in 20-mo-old mice were significantly declined compared with 2- and 16-mo-old mice (**A-B**). The reduction in protein expression of PGP9.5 (**C**) and HuC/D (**D**) was observed in aging mice consistent with morphological results. Densitometric analysis of protein expressions normalized to α-Tubulin. Statistical analysis was performed using one-way analysis of variance and data were represented as mean ± SD, statistical significance is: (B-D) ## *P* < 0.01, ### *P* < 0.001 compared with 2-mo-old group; ** *P* < 0.01, *** *P* < 0.001 compared with 16-mo-old group; n=5 mice per group. Abbreviation: Co, colon.

Furthermore, the total enteric neurons were evaluated by pan-neuronal markers protein gene product 9.5 (PGP9.5) and human neuronal protein HuC/HuD (HuC/D) immunostaining and western blotting, as observed in young (2-mo-old) and old (16-, 20-mo-old) mouse colon. PGP9.5-positive MP including neurons and processes, as well as nerve fibers were well exhibited via immunoﬂuorescence staining, and quantification of PGP9.5(+) area per field did not show significant reduction until 20 mo, matching results obtained from the test of ChAT- and NOS-positive MP (Fig. 5A-B). Western blot analysis on PGP9.5 (Fig. 5C) and HuC/D (Fig. 5D) protein in colonic muscle layer containing MP showed the similar results.

### Cx43 expression in the mouse GI tract is reduced during aging

**Figure 6:**
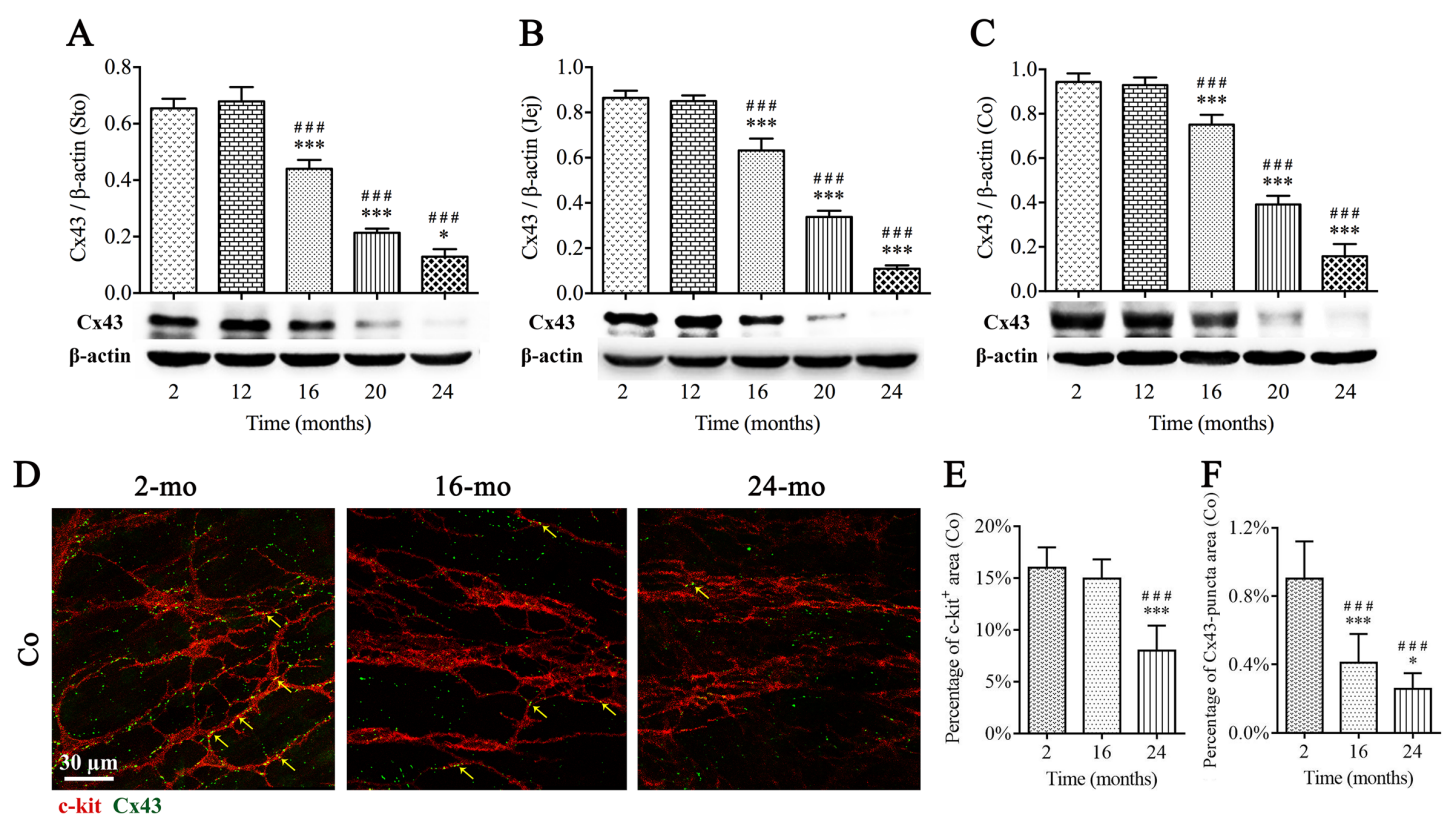
**The decrease of Cx43 protein expression in the mouse GI tract with aging.** Expressions of Cx43 within GI longitudinal muscle layer of 2-, 12-, 16-, 20- and 24-mo-old mice were examined by western blotting, and the decline in Cx43 expression was seen from 16 mo in all three organs (**A-C**). The densitometric analysis of protein expressions normalized to β-actin. Confocal images (**D**) of double immunofluorescence labelling for Cx43 (green) and c-kit (red) displayed that Cx43-puncta distributed throughout MP of colon, and some co-localized with ICCs (arrows), and in 16- and 24-mo-old mice, Cx43-puncta per field was significantly sparse compared with 2-mo-old mice, prior to the reduction in c-kit-positive area in 24-mo-old mice (**D-F**). Statistical analysis was performed using one-way analysis of variance and data were represented as mean ± SD, statistical significance is: ### *P* < 0.001 compared with 2-mo-old group; * *P* < 0.05, *** *P* < 0.001 compared with previous group; n=5 mice per group. Abbreviation: Sto, stomach; Jej, jejunum; Co, colon.

Gap junctions are important structures for cellular communication and electrical transmission. To clarify whether GI motility dysfunction in aged mice is related to the defect of gap junction structures, Cx43, as the main gap junction protein, was examined by western blotting and immunoﬂuorescence staining in mice of different ages, respectively. Importantly, expressions of Cx43 in stomach (Fig. 6A), jejunum (Fig. 6B) and colon (Fig. 6C) showed a clear tendency of decrease in elderly mice (16-, 20- and 24-mo-old). Moreover, a double‐immunofluorescence labelling of c-kit and Cx43 was presented in Fig. 6D. The Cx43 immunopositive product (green) appeared as plaques, and c-kit-positive staining (red) included cell bodies of ICCs and their processes. The high density of Cx43-puncta distributed throughout colonic MP of 2-mo-old mice, and some co-localized with ICCs (arrows). Cx43-puncta was sparse in 16- and 24-mo-old mice, whereas c-kit-positive area was not significantly diminished until 24 mo (Fig. 6D-F), suggesting Cx43 decline prior to ICCs loss.

### ICCs, enteric neurons and Cx43 are lost in the geriatric colon

**Figure 7:**
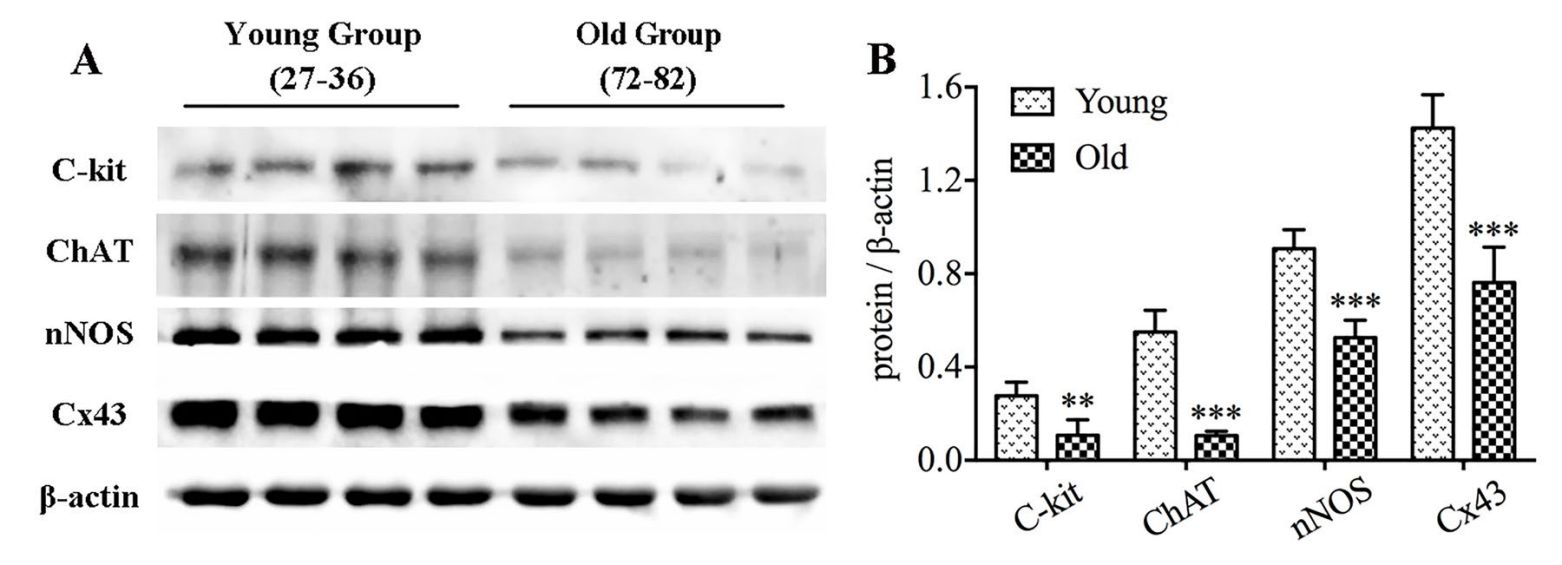
**The decrease in protein expressions of c-kit, ChAT, nNOS and Cx43 in the colon of elderly humans.** Expressions of c-kit, ChAT, nNOS and Cx43 proteins in colonic muscle layers detached from youth and older adults were examined by western blotting (n=4 per group), and similar reduction was observed (**A-B**). The densitometric analysis of protein expressions normalized to β-actin. Statistical analysis was performed using Student’s *t*-test and data were represented as mean ± SD, statistical significance is: ** *P* < 0.01, *** *P* < 0.001 compared with young group.

To clarify differences of ICCs, enteric neurons and Cx43 between young and old cohorts, we collected 8 samples of normal colonic muscle layer, four each from young group (27-36 years old) and old group (72-82 years old). Age-related changes in protein expressions were measured by western blotting (Fig. 7). It was clearly seen that c-kit and ChAT were significantly reduced in old people. Simultaneously, nNOS and Cx43 were also shown to be down-expressed in elderly samples.

### Increased inflammation and apoptosis are present in the aged colon

**Figure 8:**
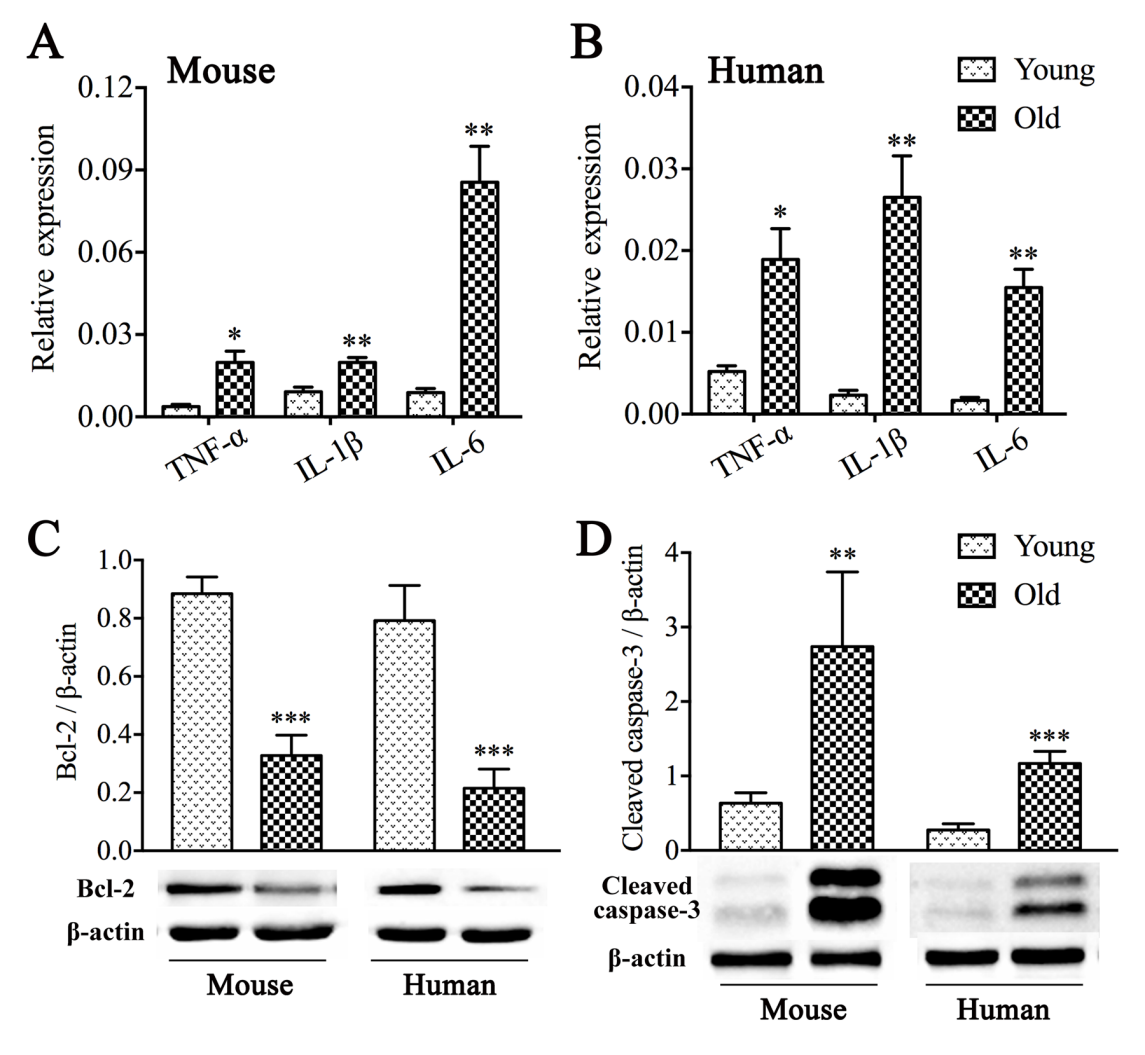
**Inflammation and apoptosis in the muscle layer from aged colon.** Both aged mice (**A**) and old people (**B**) exhibited elevated mRNA expression levels of classic pro-inflammatory cytokines including TNF-α, IL-1β and IL-6 compared with those from young. GAPDH was used as internal control. Reduced expression of Bcl-2 (**C**) and a rise in expression of cleaved caspase-3 (**D**) were revealed by western blot analysis. The densitometric analysis of protein expressions normalized to β-actin. Statistical analysis was performed using Student’s *t*-test and data were represented as mean ± SD, statistical significance is: * *P* < 0.05, ** *P* < 0.01, *** *P* < 0.001 compared with young group; n=5 mice or 4 human samples per group.

In order to explore mechanisms involved in aging that cause the loss of ICCs and enteric neurons, we performed mRNA expression analysis of classic pro-inflammatory cytokines including tumour necrosis factor-α (TNF-α), interleukin (IL)-1β and IL-6. A statistically significant rise in overall pro-inflammatory cytokines was found both in aged mice (Fig. 8A) and geriatric people (Fig. 8B) compared with young. We further investigated the expression of key proteins related apoptosis in the colonic muscle layer. As shown in Fig. 8C-D, decreased B-cell lymphoma-2 (Bcl-2) and increased cleaved caspase-3 were observed in old compared to young group.

## DISCUSSION

GI dysmotility is the most common disease in older folks and leads to unfavorable outcomes, illumination of its pathogenesis and progression will help prevention and early intervention for elderly patients with GI motility disorders. Our results of increased small intestinal length and decreased ink propulsion rate signally with aging indicated that old age brought weakened intestinal tension, slowed bowel movement and retarded intestinal propulsion. Previous investigations emphasized that aging was accompanied by loss of ICCs and enteric neurons throughout the gut, which brought on the impaired GI motility [9,14,25,26]. As expected, in colonic muscle layer of elderly people over age 70, we observed the lower level of c-kit protein and disordered expression of key components participated in neurotransmitter synthesis such as ChAT and nNOS. Nevertheless, the onset time of decrease in ICCs and enteric neurons is still incompletely clarified. The present study is a detailed description of the degenerative pattern of ICCs, enteric neurons and Cx43 expression in murine GI tract from 2 to 24 mo, and the alterations are also compared and discussed in the whole digestive tract including stomach, jejunum and colon during aging.

Previous studies were mainly concerned with gastric motility during aging, such as the weakening in gastric relaxation and the reduction in hunger ratings [27,28]. Our results supported that this pathophysiological change might cause by disruption and loss of ICCs, cholinergic and nitrergic neurons in the mouse stomach since 16 mo. Although alterations of ICCs and enteric neurons were observed in the whole digestive tract, stomach was the first organ badly affected in the progression of aging, which might explain the clinical phenomenon usually appeared in older adults, like bloating and indigestion. Madsen and Graff [29] reported that transit of a radiolabeled meal through the upper gut occurred at a comparable rate in the healthy elderly and the young, but is slower through the colon in the elderly. Our results highlighted a possible reason behind it that the degradation level of ENS neurons fell sharply in colon than in stomach and small intestine, and was the lowest in colon. Noteworthy, in the present study, agedness took mild effect on ICCs without causing visible degenerative changes in colon till 24 mo, in which there was the obvious ICCs loss. Therefore, it was not surprising that constipation is the most common GI complaint in the elderly compared with the young and middle-aged. Nevertheless, it is worth reminding that ICCs and enteric neurons changed synchronously in stomach from 16 mo of age, which earlier than small intestine and colon, also manifesting that stomach is the first organ to be injured during aging among the whole alimentary canal, even though the symptoms of gastric dysfunction may be not severe. Moreover, our results made it clear that the reduction of ICCs was from 16 mo in stomach, 20 mo in jejunum and 24 mo in colon. In spite of the role of ICCs involving in the propagation of propulsive activities, Gomez-Pinilla *et al*. [9] argued that decrease in ICCs with aging in human did not cause serious GI motility dysfunction, hinting that other factors, including interactions between ENS and ICCs or ENS and smooth muscle, might also impact GI motility function.

In fact, the alteration of subpopulation of neurons in the elderly is somewhat controversial [15]. Kim and his research team [30,31] displayed a decrease of nitrergic neurons in colonic muscle layer and gastric mucosa of aged F344 rats. In contrast, some reports showed cholinergic neurons were selectively vulnerable, whereas nitrergic neurons were spared during aging [32–34]. While our results demonstrated a notable dwindling number of NOS(+) neurons in the MP with advanced age, and ChAT(+) neurons presented the same reduced tendency to distribution, which was similar to a recent report that demonstrated the reduction in the density of Hu(+) neurons within myenteric ganglia in old mice [14]. Overall, considerable evidence showed that the ENS could be a sensitive matter of GI function, and our results of the reduction in total enteric neurons detected by PGP9.5 and HuC/D also increased the reliability of this proposition. In addition, we noticed that the ENS function might be easily and negatively influenced beyond visible numeral and network structural abnormality of enteric neurons, because the small intestinal transit rate was declined from 16 mo, while the disturbance of both ChAT(+) and NOS(+) neurons did not appear until 20 mo. It is known that obstacle to the neurotransmitter synthesis is reversible, so this result may provide a critical window of opportunity for early intervention to reverse or improve the GI motility dysfunction during aging in clinic.

A more meaningful result in our study was that Cx43 protein expression was significantly reduced over time, which occurred prior to the disruption and loss of ICCs and enteric neurons. The downward trend of Cx43 expression started from 16 mo in both stomach and intestine, which was consistent with the pattern of small intestinal transit. Thus, we speculated that the decline in expression of Cx43 would attenuate cellular communication and signal transduction between ICCs and/or SMCs, in turn led to abnormal action potential conduction. Therefore, we performed double-labelling immunofluorescence with Cx43 and c-kit (recognizing ICCs), the result indicated that decreased Cx43-puncta showed up before morphologic alterations of ICCs, raising the intriguing possibility that Cx43 might be a contributing factor resulting in GI motility dysfunction during aging, and hence, maintaining the normal expression of Cx43 in GI tract would also be an attractive treatment to relieve early age-related symptoms clinically.

Although the mechanisms causing loss of ICCs and enteric neurons during aging remain poorly understood, a growing body of evidence suggests that inflammation appears to be involved [35,36]. In animal models of colitis [37] and aged mice [14], inflammation has been shown to cause the loss of myenteric neurons. Notably, chronic low-grade inflammation, termed “inflamm-aging”, has been observed in other tissues and organs with aging [38]. Our study demonstrated that elevated pro-inflammatory cytokines including TNF-α, IL-1β and IL-6 in the muscle layer containing MP and ICCs in old mice, similar to elderly population, paralleled myenteric neuronal loss and functional delay in intestinal transit, mirroring those seen in aged small intestine [14]. Given the pro-apoptotic effect of pro-inflammatory factors, particularly TNF-α and IL-6, we further evaluated the expression of apoptosis-related proteins. As expected, there was a significant change in Bcl-2 and cleaved caspase-3 in old group compared to young. These results illustrated that age-related inflammatory state and excessive apoptosis might be associated with loss of ICCs and enteric neurons, and raised the possibility that anti-inflammatory intervention at early stage might prevent ICCs and neurons loss and even improve intestinal transit. Further experiments aimed at elucidating the detailed mechanism are needed.

In conclusion, we demonstrated that reduction in numbers of ICCs and enteric neurons first appeared in stomach and then in intestine, suggesting that stomach was the first impaired organ during aging process and this might explain common gastric motor dysfunction in the elderly. Importantly, the decrease in Cx43 expression occurred prior to the decline in ICCs and ENS neurons in aged GI tract, was likely to be involved in mediating earlier dysmotility, providing possible therapeutic time window for beneficial intervention for geriatric patients with GI dysfunction in clinic. Furthermore, with time, “inflammaging” in the ENS microenvironment would contribute to ICCs and neurons loss and development of GI disorders.

## MATERIALS AND METHODS

### Animals

A total of 60 C57BL/6 mice of either gender were purchased from Capital Medical University and divided into five groups according to age: 2-, 12-, 16-, 20- and 24-mo-old, to simulate life phases at approximately 20, 40, 50, 60 and 70 years of age in human [39]. Mice were housed in a controlled environment under a 12 h light/dark cycle at 22 ± 2 °C, 55 ± 5% humidity with access to food and water ad libitum. All mice were sacrificed by appropriate anesthesia and cervical dislocation prior to sample collection. The gastric body, the middle part of the jejunum and colon were surgically removed from mice and used for further experiments. All animal procedures were carried out strictly under protocols approved by the Animal Care and Use Committee of Capital Medical University (Permit Number AEEI-2015-143, 17 October 2015). Every effort was made to minimize the number of animals used as well as their suffering.

### Human tissue samples

The clinical tissue samples, including 8 normal colonic muscle layers from different colorectal cancer patients, were collected immediately after surgical resection prior to any other therapeutic intervention at Beijing Friendship Hospital, Capital Medical University (Beijing, China). All patients were chemotherapy and radiation therapy naive. The young group (n=4) was 27 to 36 years old, and the elderly group (n=4) was 72 to 82 years old. The study protocol was approved by the Clinical Research Ethics Committee of Beijing Friendship Hospital, Capital Medical University (Permit Number 2015SY12, 9 March 2015). Tissue samples were stored at -80 °C immediately after collection.

### Intestinal transit measurement

Mice were fasted for 16 h prior to experiments, and then received an intragastric administration of 0.2 mL carbonic ink to evaluate the intestinal transit rate. Thirty minutes later, mice were sacrificed, and the whole intestinal tract, from the cardia to the terminal rectum, was removed. Without applying tension, length of the small intestine as well as ink propulsion distance were measured. The intestinal transit rate was calculated by using the following formula:

ink propulsion rate (%) = migration distance of ink (cm) / small intestine length (cm) × 100%.

### Whole-mount preparation

The gastric body, jejunum and colon were fully washed with pre-cooled phosphate buffered saline (PBS, 0.01 M, pH 7.4) at 4 °C, then inflated with 100% acetone for c-kit, Cx43 and NADPH-d staining or 4% paraformaldehyde for ChAT and PGP9.5 staining, and immersed in the same ﬁxative at 4 °C for 24 h. After washing with PBS, mucosa and submucosa were removed, the longitudinal muscle with adherent MP was carefully dissected with the aid of a dissecting microscope (Leica S6E, Germany), then the whole-mount preparation with an area of 0.25 cm^2^ (0.5 × 0.5 cm) was obtained.

### Immunofluorescence staining

Whole-mount preparations were washed in PBS containing 0.3% Triton X-100 3 times for 10 min each. Non-specific binding was blocked with 1% bovine serum albumin (BSA, Sigma-Aldrich, USA) for 1 h, followed by primary (overnight at 4 °C or 24 h at 25 °C) and secondary antibodies (1 hour at 25 °C) as specified in Table 1. Negative-control sections were incubated in solutions lacking the primary antibody.

**Table 1:** The details of antibodies used in the experiments.

**Antibody**	**Source**	**Dilution**
**For immunofluorescence staining**		
c-kit	eBioscience, Cat.No.14-1172	1:200
ChAT	Novus, Cat.No.NBP1-30052	1:100
UCHL1/PGP9.5	ProteinTech, Cat.No.14730-1-AP	1:200
Cx43	Abcam, Cat.No.ab11370	1:1000
Goat anti-Rat IgG (Cyanine3)	Invitrogen, Cat.No.A-10522	1:200
Goat anti-Rat IgG (TRITC)	ProteinTech, Cat.No.SA00007-7	1:100
Donkey anti-Goat IgG (Alexa Fluor 488)	Invitrogen, Cat.No.A-11055	1:400
Goat anti-Rabbit IgG (Alexa Fluor 488)	Invitrogen, Cat.No.A-11034	1:200
**For western blot analysis**		
c-kit	CST, Cat.No.3074	1:1000
ChAT	Novus, Cat.No.NBP1-30052	1:2000
nNOS	Invitrogen, Cat.No.PA1-033	1:1000
HuC/HuD	Invitrogen, Cat.No.A-21271	1:1000
UCHL1/PGP9.5	ProteinTech, Cat.No.14730-1-AP	1:1000
Cx43	Abcam, Cat.No.ab11370	1:8000
Bcl-2	CST, Cat.No.3498	1:1000
cleaved-caspase-3	CST, Cat.No.9664	1:1000
α-Tubulin	ProteinTech, Cat.No.11224-1-AP	1:1000
β-actin	Santa Cruz, Cat.No.sc-47778	1:2000
Goat anti-Rabbit IgG (HRP)	Abcam, Cat.No.ab6792	1:2000
Goat anti-Mouse IgG (HRP)	Santa Cruz, Cat.No.sc-2005	1:2000
Donkey anti-Goat IgG (HRP)	Abcam, Cat.No.ab97110	1:2000

### NADPH-d staining

NADPH-d activity was detected histochemically to show the neurons containing NOS in the GI tract. Whole-mount preparations were washed in PBS, and then incubated in phosphate buffer (0.1 M, pH 7.4) containing 0.3% Triton X-100, 0.5 mg/mL nitroblue tetrazolium (Cat.No.N6876, Sigma-Aldrich, USA) and 1.0 mg/mL β-NADPH (Cat.No.N7505, Sigma-Aldrich, USA) for 1 h at 37 °C. The reaction was halted by placing the sample in PBS.

### Western blot analysis

The entire muscle layer of alimentary tract was detached from mucosa and submucosa using a dissecting microscope and stored in liquid nitrogen for western blot analysis. Total proteins were extracted from tissues using the RIPA lysis buffer (Cat.No.C1053, Applygen, China) containing protease inhibitor cocktail (Cat.No.P8340, Sigma-Aldrich, USA) and phosphatase inhibitor cocktail (Cat.No.P5726, Sigma-Aldrich, USA). The protein concentrations were determined by NanoDrop 2000c spectrophotometer (Thermo Scientific, USA) using BCA protein assay kit (Cat.No.P1511, Applygen, China). Equal amounts of total protein from each sample were electrophoresed on SDS–PAGE and transferred to a nitrocellulose membrane. After blocking with Tris-buffered saline containing 0.05% Tween-20 (TBST) and 5% non-fat dry milk or 5% BSA for 1 h, membranes were then orderly incubated with primary and secondary antibodies as specified in Table 1. The protein bands were detected using enhanced chemiluminescence (Thermo Scientific, USA) and viewed in Fusion FX Vilber Lourmat (France). The housekeeping gene β-actin was used as an internal control.

### RNA isolation and quantitative real-time PCR

Total RNA was extracted from tissues with TRIzol reagent (Cat.No.15596026, Life Technologies, USA) and converted to cDNA using All-In-One RT MasterMix (Cat. No. G486, ABM, Canada). Twenty microliter reactions were incubated in a Veriti 96-well Thermal Cycler (Applied Biosystems, USA) for 30 min at 42 °C and 5 min at 85 °C. Quantitative real-time PCR was performed on an ABI 7500 real-time PCR system (Life Technologies, USA) using Maxima SYBR Green/ROX qPCR Master Mix (Cat.No.K0222, Thermo Scientific, USA). For analysis, each gene was normalized to the housekeeping gene *Glyceraldehyde-3-phosphate dehydrogenase* (GAPDH). The following primers are listed in Table 2. Twenty-five microliter reactions were incubated at 95 °C for 10 min, followed by 40 cycles at 95 °C for 10 s, 60 °C for 10 s, and 72 °C for 40 s. Relative fold change of gene was calculated using the 2^−ΔΔCt^ method.

**Table 2:** Primer sequences used in the study.

**Gene name**	**F/R^a^**	**Sequence 5’-3’**
Mouse TNF-α	F	GATTATGGCTCAGGGTCCAA
	R	GCTCCAGTGAATTCGGAAAG
Human TNF-α	F	TCCTTCAGACACCCTCAACC
	R	AGGCCCCAGTTTGAATTCTT
Mouse IL-1β	F	TGCCACCTTTTGACAGTGATG
	R	TGATGTGCTGCTGCGAGATT
Human IL-1β	F	GGGCCTCAAGGAAAAGAATC
	R	TTCTGCTTGAGAGGTGCTGA
Mouse IL-6	F	CCGGAGAGGAGACTTCACAG
	R	CAGAATTGCCATTGCACAAC
Human IL-6	F	TACCCCCAGGAGAAGATTCC
	R	TTTTCTGCCAGTGCCTCTTT
Mouse GAPDH	F	TGCACCACCAACTGCTTAG
	R	GGATGCAGGGATGATGTTC
Human GAPDH	F	AGAAGGCTGGGGCTCATTTG
	R	AGGGGCCATCCACAGTCTTC

^a^ F, forward primer; R, reverse primer.

### Quantiﬁcation of cell numbers and data analysis

Images were acquired with a fluorescence microscope (Nikon 90i, Japan), confocal microscope (Leica DM6000 CS, Germany) equipped with confocal spectral scanning system (Leica TCS SP8) or light microscope (Leica DM LB2) and analyzed using Image-Pro Plus 6.0 software (Media Cybernetics Inc., USA). Data analysis was assessed in randomly chosen non-overlapping fields-of-view. Four fields in each whole-mount preparation were randomly taken, 5 preparations of each mouse and 5 mice per group were used. Quantifications of ICCs and nitrergic neurons were performed by counting the number of positive cells of all fields from each whole-mount preparation, and the cell density was determined by the total area of the ﬁelds. The cell morphology of inclusion criteria, including fusiform or stellate-shaped ICCs with slender processes and nitrergic neurons colored purple-blue, was used for identification during counting. The positive areas of cholinergic neurons, PGP9.5(+), c-kit(+) neurons and their nerve fibers as well as Cx43-puncta were conducted derived by dividing the total areas of the fields.

### Statistics

Obtained data were analyzed using one-way analysis of variance for multiple groups and Student’s *t*-test for two groups with SPSS 23.0 software (IBM Corporation, USA). A *P* value < 0.05 was considered to be statistically significant.
